# Complete Genome Sequence of a Tomato-Infecting Tomato Mottle Mosaic Virus in New York

**DOI:** 10.1128/genomeA.01523-15

**Published:** 2015-12-23

**Authors:** Chellappan Padmanabhan, Yi Zheng, Rugang Li, Gregory B. Martin, Zhangjun Fei, Kai-Shu Ling

**Affiliations:** a USDA-Agricultural Research Service, U.S. Vegetable Laboratory, Charleston, South Carolina, USA; b Boyce Thompson Institute for Plant Research, Ithaca, New York, USA; c Section of Plant Pathology and Plant-Microbe Biology, School of Integrative Plant Science, Cornell University, Ithaca, New York, USA; d USDA-Agricultural Research Service, Robert W. Holley Center for Agriculture and Health, Ithaca, New York, USA

## Abstract

The complete genome sequence of an isolate of tomato mottle mosaic virus (ToMMV) infecting tomatoes in New York was obtained using small RNA (sRNA) deep sequencing. ToMMV_NY-13 shared 99% sequence identity with isolates from Mexico and Florida. Broader distribution of this emerging virus is a cause for concern to the tomato industry.

## GENOME ANNOUNCEMENT

For many years, tobacco mosaic virus (TMV) and tomato mosaic virus (ToMV) have been two of the most common recognizable tobamoviruses infecting tomatoes. These seed-borne and mechanically transmitted viruses are managed through the breeding of tobamovirus-resistant tomato cultivars and in the use of certified, virus-tested seed lots. However, a novel tobamovirus, tomato mottle mosaic virus (ToMMV), was first identified in tomatoes in Mexico in 2013 (1) and subsequently detected in tomatoes in Florida, USA (2, 3). ToMMV has also been reported to infect pepper (GenBank accession number KJ605653) in China (4). Based on sequence similarity analysis, this virus is likely present in several other countries, including Brazil (AF411922), Iran (HQ593616, JX112024, JX112025, JX121570, JX121574, JX121575, and JX121576), and Israel (KP861747 and KP861748). ToMMV is a tentative member of the genus *Tobamovirus* in the family *Virgaviridae* (1). In the summer of 2013, experimental tomato plants in greenhouses in upstate New York were surveyed for possible virus infection using small RNA (sRNA) deep-sequencing technology (5, 6). Total plant RNA was extracted from a bulked leaf tissue sample using TRIzol reagent (Invitrogen, USA). Small RNA libraries were prepared as described (7) and sequenced using an Illumina HiSeq 2000. Small RNA sequence reads were assembled after subtraction of the host plant sRNAs, and virus identification was carried out using a bioinformatics pipeline (7). A contig containing a full-length genome sequence for ToMMV was obtained and its sequence validated through reverse transcription (RT)-PCR amplicons generated using ToMMV-specific primers. The 3′ end was validated by using 3′ rapid amplification of cDNA ends (RACE) followed by Sanger sequencing. The complete genome sequence of the identified ToMMV_NY-13 isolate consisted of 6,398 nucleotides. BLASTn searches to the NCBI databases revealed that ToMMV_NY-13 shared the highest sequence identity (99%) with existing complete sequences of ToMMV isolates from Mexico (KF477193) and Florida (KP202857). The genome organization of the newly identified ToMMV_NY-13 isolate consisted of four open reading frames (ORFs) of a typical tobamovirus. The first ORF encoded a 126-kDa protein containing methyl-transferase and helicase domains. The second ORF encoded a 183-kDa read-through protein containing RNA-dependent RNA polymerase (RdRp), in addition to the methyltransferase and helicase domains. The movement protein (MP) in the third ORF and the coat protein (CP) in the fourth ORF were expressed through their respective subgenomic RNAs. The identification of ToMMV_NY-13 in New York State indicates a broader distribution of this emerging virus in the United States than was known previously (i.e., from Florida in the Southeast to New York in the Northeast). Additional surveying is necessary to determine the geographical distribution of this emerging virus in the United States and around the world. Further study of the ToMMV genome sequence and the genetic diversity of this virus is needed to allow the development of molecular-based detection methods and disease management strategies.

### Nucleotide sequence accession number.

The complete genome sequence of ToMMV_NY-13 has been deposited in GenBank under the accession number KT810183.
